# Diversity and transmission of koala retrovirus: a case study in three captive koala populations

**DOI:** 10.1038/s41598-022-18939-6

**Published:** 2022-09-22

**Authors:** Briony A. Joyce, Michaela D. J. Blyton, Stephen D. Johnston, William D. Meikle, Kimberly Vinette Herrin, Claire Madden, Paul R. Young, Keith J. Chappell

**Affiliations:** 1 School of Chemistry and Molecular Biosciences, The University of Queensland, St. Lucia, QLD 4072 Australia; 2 Australian Institute for Bioengineering and Nanotechnology, The University of Queensland, St. Lucia, QLD 4072 Australia; 3 School of Agriculture and Food Sciences, The University of Queensland, Gatton, QLD 4343 Australia; 4 WILD LIFE Sydney Zoo, Darling Harbour, Sydney, NSW 2000 Australia; 5 Taronga Wildlife Hospital, Taronga Zoo, Mosman, NSW 2088 Australia; 6 Sea World and Paradise Country, Main Beach, QLD 4217 Australia

**Keywords:** Virology, Viral transmission

## Abstract

Koala retrovirus is a recently endogenized retrovirus associated with the onset of neoplasia and infectious disease in koalas. There are currently twelve described KoRV subtypes (KoRV-A to I, K–M), most of which were identified through recently implemented deep sequencing methods which reveal an animals’ overall KoRV profile. This approach has primarily been carried out on wild koala populations around Australia, with few investigations into the whole-population KoRV profile of captive koala colonies to date. This study conducted deep sequencing on 64 captive koalas of known pedigree, housed in three institutions from New South Wales and South-East Queensland, to provide a detailed analysis of KoRV genetic diversity and transmission. The final dataset included 93 unique KoRV sequences and the first detection of KoRV-E within Australian koala populations. Our analysis suggests that exogenous transmission of KoRV-A, B, D, I and K primarily occurs between dam and joey. Detection of KoRV-D in a neonate sample raises the possibility of this transmission occurring in utero. Overall, the prevalence and abundance of KoRV subtypes was found to vary considerably between captive populations, likely due to their different histories of animal acquisition. Together these findings highlight the importance of KoRV profiling for captive koalas, in particular females, who play a primary role in KoRV exogenous transmission.

## Introduction

Koala retrovirus (KoRV) is a gammaretrovirus, closely related to gibbon ape leukemia virus (GALV) and feline leukemia virus, that presents in both wild and captive koalas^1^. It is ubiquitous in koalas in the northern states of Queensland (QLD) and New South Wales (NSW), however, a lower prevalence is observed in the southern regions (states of Victoria and South Australia) of Australia^1–11^. Uniquely, KoRV is one of the only viruses that presents in both exogenous and endogenous forms, with endogenization (permanent integration into the host genome) estimated to have occurred less than 50,000 years ago^12,13^. As a permanent fixture of the koala genome, these retroviral elements have helped shape koala evolution. Both exogenous and endogenous forms of KoRV have putatively been associated with the onset of koala disease, including neoplasia, leukemia, lymphoma, myelodysplasia, and a range of infections including chlamydiosis, a bacterial pathogen causing koala morbidity, infertility and mortality^7,14–18^. Despite this, there has been little investigation into population-wide KoRV profiling of captive koala colonies to identify those most at threat and those with the greatest transmission potential.

There are currently 12 described KoRV subtypes (KoRV-A to I, K–M), each possessing a cluster of closely related sequences within the receptor binding domain (RBD) of the envelope glycoprotein^1,3,4,11,19–22^. KoRV-A was the first subtype identified and was later found to have endogenized into the koala genome where it has permanently incorporated at multiple sites, although the location of these sites varies between individuals^1^. This endogenized sequence is, therefore, vertically transmitted through to the progeny from their dam and/or sire. The functional, endogenous KoRV-A sequence (AF151794) has been detected in all analyzed wild koalas residing in the northern regions of eastern-Australia (QLD and NSW) at copy numbers of around 70 per genome^23^. However, the genomes of koalas found in the southern parts of Australia, appear to only possess partial variants of this KoRV sequence at far lower copy numbers^3–5,8,11,24^. Exogenously transmitted functional KoRV-A is present in both northern and southern koala populations, however, it appears to be present at a lower prevalence within southern koalas^3–5,8,11,24^.

The remaining 11 KoRV subtypes (B–I, K–M) have only been discovered in the past 10 years through analysis of wild koalas and captive colonies around the world^3,4,11,19–22^. In comparison to KoRV-A, these subtypes are found at a considerably lower prevalence and within koala abundance, with their detection in only a proportion of the animals analyzed to date^3–5,10,11,25,26^. Subtype variation is seen both between animals and between populations, however, of these nine subtypes, KoRV-B (also previously referred to as J) and D are consistently found to be the most prevalent^3–6^. Unlike KoRV-A, subtypes B–M are currently only believed to transmit exogenously, with no reported evidence of endogenization to date. Transmission of these exogenous subtypes has been found to occur primarily between dam and joey, in the early stages of development^3^. This finding has been observed in several captive populations around the world^19,21,24,27,28^, however the only statistical test of this has been documented in two captive populations from South-East QLD^3^.

The vast increase in KoRV diversity detected over the last decade has been largely attributable to the implementation of next generation sequencing methods. This deep sequencing approach captures all KoRV diversity within the RBD of the *env* gene as opposed to isolating individual KoRV subtypes through PCR based methods which were previously used in this field^10,20,21,24^. The first implementation of this method by Chappell et al.^4^ resulted in the identification of four novel KoRV subtypes (F–I) in 18 wild koalas, expanding our known KoRV diversity at the time by two-fold. Since its initial application six years ago, deep sequencing has been carried out on several koala populations around Australia to determine the full KoRV profile of individual animals and whole koala populations. However, these have mainly focused on wild koala populations with few investigations into captive koala colonies^3,5,6,11,28^. Captive koalas suffer high rates of neoplasia, including both leukemia and lymphoma, which reduces their life span, making it a key issue for captive management^29^. Knowing the KoRV profiles of captive animals and its transmission route will therefore help inform appropriate breeding and housing strategies.

In this study, we sought to determine the KoRV genetic profiles of captive koalas housed in three Australian institutions (*n* = 64). Key genetic differences were highlighted between these colonies, providing insight into population specific diversity. Furthermore, KoRV sequence sharing between dam-joey, sire-joey and mating koala pairs were directly compared to infer exogenous viral transmission routes within these populations. The results from this study broaden our understanding of KoRV diversity and variability among captive koala populations and provide further support for the dam-joey transmission of exogenous KoRV subtypes.

## Materials and methods

### Blood sample collection and processing

A total of 64 (42 female, 22 male) clinically healthy, Australian captive koalas were included in this study, sampled from one population in South-East Queensland: colony C (*n* = 33), and two populations in New South Wales: colony D (*n* = 14) and colony E (*n* = 17). At all zoos, koalas were reared in an environment which resembled the koala natural habitat and were provided an ad libitum supply of a variety of suitable eucalyptus leaves. From each conscious koala, ~ 2 mL of blood was drawn by a veterinarian during annual health examinations over a 1-year period (March 2019–February 2020) and stored on ice during transport to the laboratory. Blood samples were centrifuged for 2 min at 11,200 × *g*, after which, the blood plasma and buffy coat (~ 100 μL) fractions were separated. Proviral genomic DNA was then extracted from the buffy coat using the FavorPrep blood genomic DNA mini kit (Favorgen Biotech Corp), as per the manufacturer’s instructions.

Sampling procedures were approved by the University of Queensland Animal Ethics Committee (animal ethics number SCMB/094/18/DREAMWORLD), the Taronga Animal Ethics Committee (animal ethics numbers 4c/04/17 and 4a/02/13) and the Secretary’s Animal Care and Ethics Committee (animal ethics number CSB 19/2473). All methods were carried out in accordance with the relevant guidelines and regulations. This study is reported in accordance with ARRIVE guidelines.

### Tissue sample collection and processing

Two koala neonates and one spleen sample were collected opportunistically from colony D. Neonate samples were bisected and stored at − 80 °C with ethanol until sample processing. The spleen sample was collected during necropsy and stored at − 80 °C prior to processing. Proviral genomic DNA was extracted from both the neonate and spleen tissue samples using the FavorPrep blood genomic DNA mini kit (Favorgen Biotech Corp), as per the manufacturer’s instructions.

### Illumina sequencing and bioinformatic processing

Illumina sequencing was carried out on all gDNA samples as previously described^3^. Briefly, PCR was used to amplify a ~ 500 bp fragment encompassing the hyper-variable receptor binding domain of the KoRV envelope gene using primers containing Illumina adapter sequences^4^. Following purification and indexation, these amplicons were then sequenced (paired-end with V3 300 bp chemistry) via the MiSeq sequencing system (Illumina, USA) at the Australian Centre for Ecogenomics (The University of Queensland, Brisbane, Australia). Forward and reverse reads were then merged and filtered on size (450–600 bp) and quality (90% of sequence with a cut-off value > 20) using the Galaxy web platform on the public server at https://usegalaxy.org^30^. The quality controlled reads were then clustered de novo in QIIME 2^31^ with a similarity of 97%. Representative sequences of each QIIME cluster were then blasted against the NCBI ‘nt’ database to identify non-KoRV sequences. Those clusters only containing a single read were also omitted. The putative KoRV sequences were then aligned to the *env* nucleotide sequence of KoRV-A (GenBank accession number AF151794) using CLC Workbench 8 (CLCBio, Denmark), and those containing missense mutations, large deletions or which lacked *env* gene homology to KoRV-A were removed. KoRV subtype was then denoted to each sequence based on protein sequence homology within the hyper-variable region, in accordance with previous studies^3,4,19,20,32,33^.

### Sequence sharing analysis

The number of KoRV sequences shared between koalas of varying relation was then determined and used, in conjunction with known koala pedigrees, to infer KoRV transmission, as detailed previously^3^. For this, the sample by sequence read count table generated from QIIME was converted into a sample by sequence presence/absence matrix. The number of sequences shared between koala pairs for each subtype was then calculated using custom code in RStudio 3.5.1^34^. Using supplied koala pedigrees from all captive populations, each koala pair was classified as; unrelated (*n* = 426), dam-joey (*n* = 40), sire-joey (*n* = 16), mating partners (*n* = 17), maternally-related (up to second cousins, *n* = 100) or paternally-related (up to second cousins, *n* = 156). Maternal relatives were defined by those related through a strictly female lineage. All analyzed subtypes (exogenous A, B, D, I and K) were combined for this analysis as the extent of sharing for these subtypes independently among unrelated koalas was insufficient to allow model fitting. Due to the low incidence of KoRV-E, G and H, these subtypes were excluded.

Generalised linear mixed models following a Poisson distribution were then fitted using the MCMCglmm 2.29 package in R^35^ as previously described^3^ to determine if the extent of KoRV sequence sharing differed between koala pairs with different familial relationships. Specified model parameters were as follows: number of iterations (nitt) = 2,003,000; number of initial iterations removed (burnin) = 3000; thinning interval (thin) = 200. The endogenous KoRV-A sequence (GenBank accession number AF151794) was omitted from this analysis as it was shared among all koalas.

### Data analysis

The expected number of sequences shared for each pair type within each subtype was calculated from the fitted generalised linear mixed model parameters using the following formula: ${e}^{\text{post} \; \text{mean }+\text{ post} \; \text{mean} \; \text{of} \; \text{intercept}},$ with credible intervals also determined using: ${e}^{\text{credible} \; \text{interval} \; \text{value }+\text{ post} \; \text{ mean} \; \text{of} \; \text{intercept}}.$

## Results

### KoRV genetic diversity

Illumina *env* amplicon deep sequencing was carried out on genomic DNA isolated from the peripheral blood mononuclear cells (PBMCs) of 64 koalas housed in three captive koala colonies within Australia, one in South-East Queensland (SE QLD; colony C, *n* = 33) and two in Sydney, New South Wales (NSW; colony D, *n* = 14; colony E, *n* = 17). The hyper-variable receptor binding domain within the *env* gene was amplified which, following quality control and clustering at 97% identity, resulted in the detection of 93 unique KoRV sequences (deposited to GenBank; Table 1). Proviral genomic DNA isolated from PBMCs was used as this provides the highest detection rate for the exogenous subtypes infecting a koala compared to other sample types^3,11^.

**Table 1:** KoRV subtype diversity in captive koala populations.

Subtype	No. individuals (%)				No. unique sequences	
	Colony-C	Colony-D	Colony-E	Combined	Identified	Shared (%)
KoRV-A	33 (100%)	14 (100%)	17 (100%)	64 (100%)	25	25 (100%)
KoRV-B	8 (24.2%)	5 (29.4%)	11 (64.7%)	24 (35.8%)	13	12 (92.3%)
KoRV-C	*N.D*	*N.D*	*N.D*	*N.D*	*N.D*	*N.D*
KoRV-D	31 (93.9%)	9 (64.3%)	13 (82.4%)	53 (82.8%)	30	25 (83.3%)
KoRV-E	*N.D*	4 (23.5%)	*N.D*	4 (6%)	2	2 (100%)
KoRV-F	*N.D*	*N.D*	*N.D*	*N.D*	*N.D*	*N.D*
KoRV-G	*N.D*	*N.D*	1 (5.9%)	1 (1.5%)	2	0 (0%)
KoRV-H	*N.D*	*N.D*	2 (11.8%)	2 (3%)	4	2 (50%)
KoRV-I	10 (30.3%)	*N.D*	11 (64.7%)	21 (31.3%)	15	11 (73.3%)
KoRV-K	15 (45.5%)	*N.D*	*N.D*	15 (22.4%)	2	2 (100%)
KoRV-L	*N.D*	*N.D*	*N.D*	*N.D*	*N.D*	*N.D*
KoRV-M	*N.D*	*N.D*	*N.D*	*N.D*	*N.D*	*N.D*
TOTAL:	33	14	17	64	93	79 (84.9%)

Number of koalas possessing each subtype and the corresponding number of identified sequences for that subtype are shown for all populations. Subtypes were identified in blood genomic DNA of koalas from colony C (*n* = 33), D (*n* = 14) and E (*n* = 17). Shared sequences correspond to those present in two or more koalas. *N.D* not detected.

The read count was found to vary drastically between individuals with 19,136 KoRV reads present on average, ranging from 2280 to 64,243, after filtering and quality control. Protein alignment of the 93 in silico translated sequences to those of known KoRV subtypes led to their classification as one of eight subtypes (A, B, D, E, G, H, I and K). Consistent with other studies, KoRV-A was found to be the most prevalent subtype detected, identified in 100% of the koalas analyzed from all three populations (Table 1). This was followed closely by KoRV-D which was detected in 53 (82.8%) out of 64 koalas examined. Unsurprisingly, these two subtypes also had the greatest number of unique sequences detected with 25 KoRV-A and 30 KoRV-D sequences identified across all three institutions. KoRV-G and H were found to be the least prevalent subtypes, detected in one and two koalas, respectively. KoRV-E, G and K were found to have the least genetic diversity, with only two sequences detected for each across all KoRV-E (*n* = 4), G (*n* = 1) and K (*n* = 15) positive koalas (Table 1). KoRV-C, F, L and M were not identified within these populations.

Whilst a small proportion of sequences were only identified within a single koala, highlighting the continual within-host evolution of this virus, the majority (84.9%) of sequences were detected in two or more koalas (Table 1; Supplementary Table S2). Consistent with previous research, the full-length endogenous KoRV-A sequence (GenBank accession number AF151794) was identified within all analyzed koalas where it accounted for 98.2% of an individual’s reads on average, ranging between 80.4% and 100%. The next most within koala abundant sequence was a KoRV-B sequence (GenBank accession number ON839123) detected in 16 (25%) individuals across all three populations. This sequence accounted for 0.27% of a koala’s total KoRV reads on average, ranging between 0% and 7.4%, and 23.5% of a koala’s total KoRV-B reads on average, ranging between 0% and 99.8% (Supplementary Table S2). KoRV subtypes I and K were each found to have dominant sequences that were detected within 81% of KoRV-I (ON839175) and 100% of KoRV-K (ON839182) positive koalas, accounting on average for 41% and 97% of KoRV-I and K total reads, respectively. No dominant sequence was identified within KoRV-D positive koalas with the most prevalent sequence (ON839157) detected in less than 50% of positive koalas.

**Figure 1:**
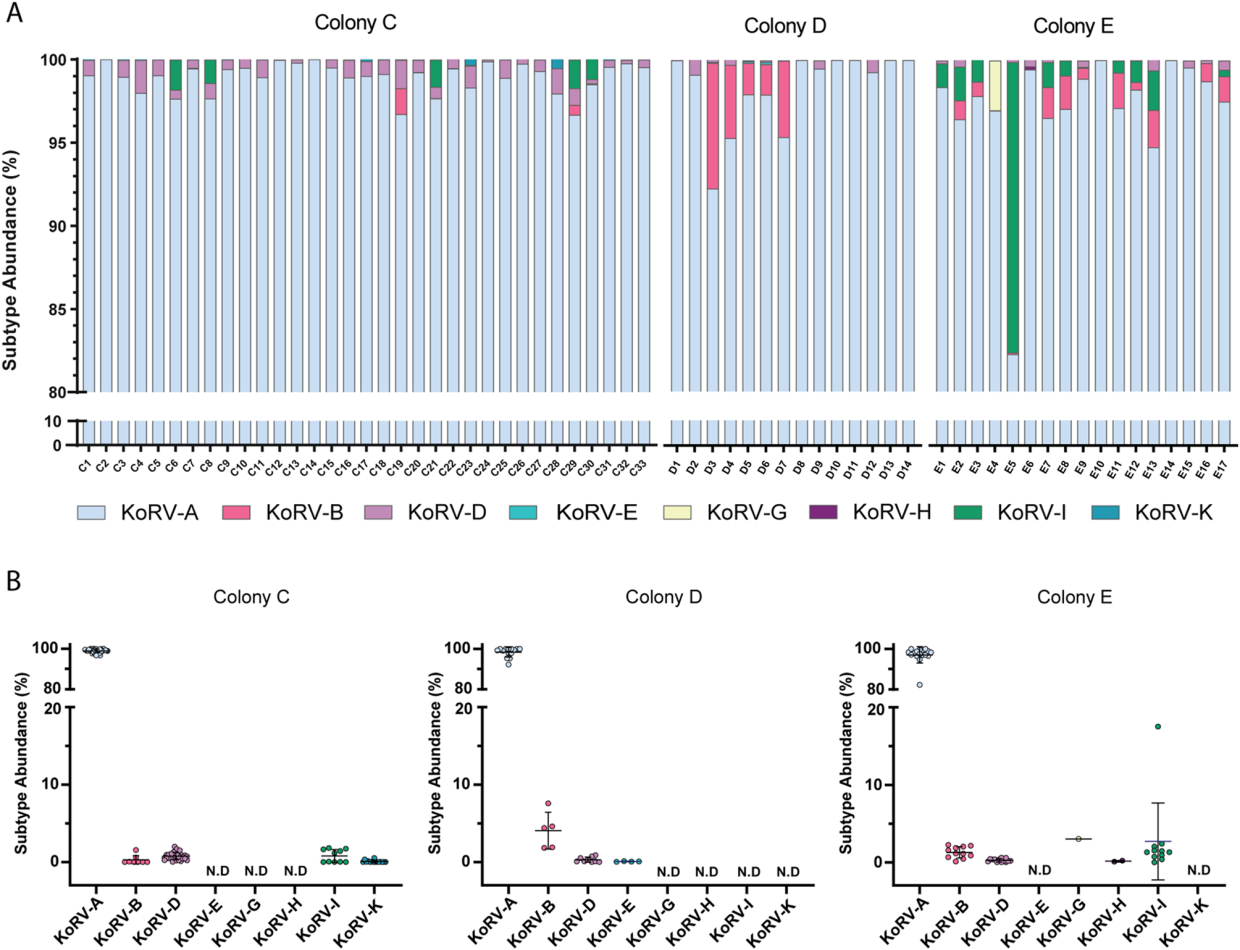
Percentage of KoRV reads grouped by subtypes. Prevalence of KoRV subtypes in genomic DNA from koalas housed at colony C (*n* = 33), colony D (*n* = 14) and colony E (*n* = 17). (**A**) Subtype abundance for each animal is shown for all populations. Colours indicate the different subtypes detected. (**B**) Percentage relative abundance for each subtype is summarized for the three populations. Each point represents an individual koala with the mean ± SD shown. *N.D* not detected.

KoRV-A was the most abundant subtype in all analyzed koalas, representing 98.3% of KoRV reads on average, ranging from 82.3% to 100%. The other subtypes were comparatively reduced, accounting for less than 20% of reads in total (Fig. 1). The number of reads attributable to each KoRV subtype for each koala is shown in Fig. 1 and Supplementary Table S1. KoRV A, B and D were the only subtypes detected in all three populations, with KoRV-A and D identified at a similar prevalence and abundance across all three. Whilst KoRV-B was found to be more prevalent within colony E (64.7% of koalas), it was most abundant in colony D where it accounted for 4% of an animal’s total KoRV reads on average (Fig. 1B). KoRV-I was found to be more prevalent in colony E (64.7% of koalas) in comparison to colony C (30.3% of koalas), however, despite an obvious outlier, it was found at a similar abundance across both populations. The other exogenous subtypes were detected variably among the populations (Fig. 1B). Overall, colony C was found to have the least genetic diversity with KoRV-A accounting for 99% of total KoRV reads on average (Fig. 1A).

At least two subtypes were detected in 55 (85.9%) of the 64 koalas analyzed, with a maximum of four subtypes identified in 23 koalas. Notably, the remaining nine koalas only had KoRV-A (Fig. 1A; Supplementary Table S1; C2, C14, D8, D10, D11, D13, D14, E10 and E14). Whilst these individuals were housed in each of the three institutions, more than 50% resided in colony D in NSW. Interestingly, this colony was found to have the least number of subtypes detected of all three populations and at a relatively low abundance (Fig. 1B).

Two neonate tissue samples from colony D were also analyzed for their KoRV genomic DNA composition. Both samples were collected opportunistically after neonates died naturally failing to make it into the pouch following birth. The endogenous KoRV-A sequence (AF151794) was found in both neonate 1 (D15) and 2 (D16), where it accounted for 99.98% and 100% of their total KoRV reads, respectively. The remaining 0.02% of KoRV reads for neonate 1 were attributable to a KoRV-D sequence. This sequence was also detected at low levels in the dam (D1), sire (D17) and both paternal grandparents (D9 and D12) of this neonate (Supplementary Fig. S1), however, not in the maternal grandmother (D13) sample. Notably, these animals were analyzed via blood (D1, D9, D12–13) and spleen tissue (D17). Both neonates shared the same dam, however the maternal grandfather and neonate 2 sire were not included in this study.

### KoRV exogenous transmission

The number of KoRV sequences shared between unrelated (*n* = 426), maternally related (m-related; *n* = 100), paternally related (p-related; *n* = 156), dam-joey (*n* = 40), sire-joey (*n* = 16) and mating partner (*n* = 17) koala pairs was compared between pair types by fitting generalised linear mixed models using the MCMCglmm 2.29 package in R^35^. A maternal lineage was defined as those related through a strictly female line. Due to the overall low sequence diversity (and consequently low number of sequences shared between koala pairs) in these populations, all KoRV subtypes were combined for analysis to provide adequate data for model fitting. This analysis did not include subtypes E, G or H, as they were detected in too few animals for meaningful comparisons to be made. The endogenous KoRV-A sequence was also omitted, as it was shared among all animals.

**Figure 2:**
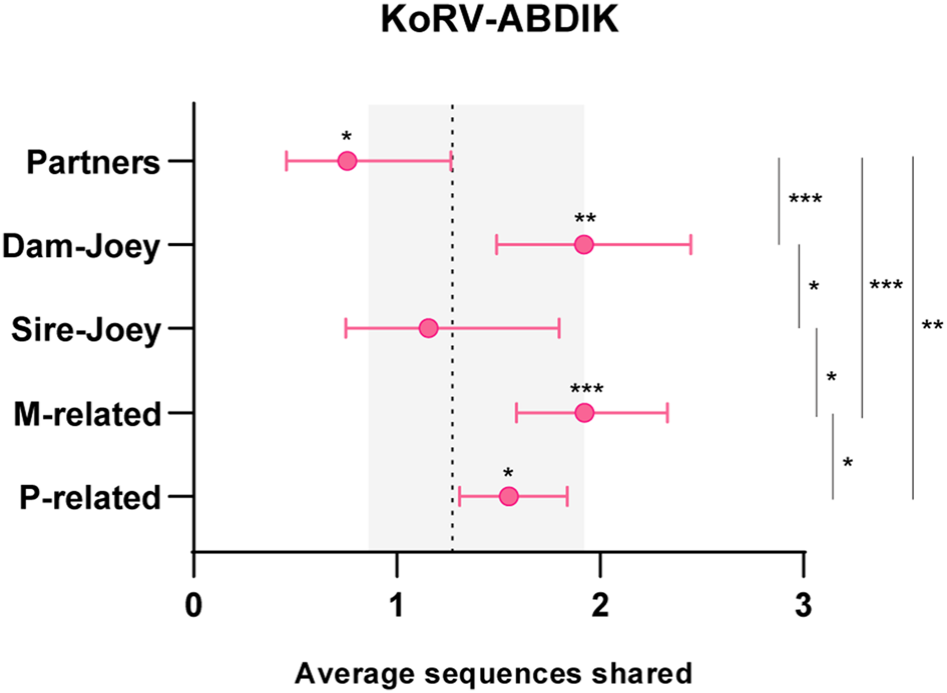
Average sequences shared with 95% credible intervals from generalised linear mixed models of subtype sharing. The expected number of shared KoRV-A, B, D, I and K sequences is shown for paternally related (P-related; *n* = 156), maternally related (M-related; *n* = 100), sire-joey (*n* = 16), dam-joey (*n* = 40) and mating partner (*n* = 17) koala pairs. Expected sharing between unrelated (*n* = 426) koalas is represented by a dashed line with 95% credible intervals highlighted in grey. Maternal relatives were defined by those related through a strictly female lineage. The original, endogenous KoRV-A sequence was omitted from this analysis. Asterisks above nodes indicate significance to the unrelated reference group. Significance between different pair groupings is shown on the right. *p < 0.05, **p < 0.01, ***p < 0.001.

Overall, dam-joey pairs were found to share significantly more sequences than unrelated and sire-joey pairs, sharing 1.9 sequences on average (95% credible interval (CI) 1.5–2.4) compared to 1.3 (95% CI 0.9–1.9) for unrelated (Fig. 2; *p* < 0.01) and 1.2 (95% CI 0.7–1.8) for sire-joey (*p* < 0.05). Similar findings were observed when comparing m-related pairs (averaged 1.9 sequences shared; 95% CI 1.6–2.3) with unrelated pairs (Fig. 2; *p* < 0.001) and p-related pairs (averaged 1.6 sequences shared; 95% CI 1.3–1.8; *p* < 0.05). Whilst significantly more sequences were shared by p-related koalas than unrelated koalas, this was not as notable as dam-joey or m-related pairs (Fig. 2; *p* < 0.05). No evidence of sexual transmission was observed, with mating partners (averaged 0.8 sequences shared; 95% CI 0.5–1.3) found to share significantly less sequences on average compared to unrelated koala pairs (Fig. 2; *p* < 0.05).

Whilst not included in the statistical modelling due to low sample size (*n* = 4), evidence of dam to joey transmission of KoRV-E was also found within colony D. In this population, KoRV-E was only identified within the offspring (D5 and D6) of two unrelated, KoRV-E positive dams (D3 and D7, respectively). Notably, however, there were no matched samples from KoRV-E positive sires in these populations.

## Discussion

Since its initial application in 2017, deep sequencing has been used to profile KoRV genetic diversity within several koala populations around Australia^3–6,11,28^. However, these studies have primarily focused on wild koala populations with minimal investigation of captive koala colonies. In this study, we analyzed KoRV genetic diversity and transmission within three Australian captive koala populations (*n* = 64), housed in both QLD and NSW, to provide insight into KoRV variability and transmission patterns among institutions. For this, deep sequencing was carried out on the receptor binding domain of the KoRV *env* gene. Utilizing known koala pedigrees, the sequences shared between related koala pairs were compared to infer exogenous transmission patterns. This study represents the first application of next generation sequencing to profile KoRV genetic diversity within captive animals housed in NSW. These findings, in combination with those from Joyce et al.^3^, provide valuable information on exogenous KoRV prevalence in captive colonies and insight into likely transmission routes.

Overall, KoRV subtype prevalence and abundance is found to vary considerably between captive populations. Consistent with previous studies, KoRV-A was found to be the most prevalent and abundant subtype in all three colonies, followed closely by KoRV-D and B^3–6^. The remaining identified subtypes were each only detected within one population, except for KoRV-I, which was found at a different prevalence in colony C (30.3% of animals) and E (64.7% of animals). Notably, KoRV-E was detected in colony D, which is the first reported instance of this subtype within Australian populations, having previously only been detected in a US zoo^21^. These differences in KoRV diversity are even more evident when compared to two analyzed captive populations from SE QLD (see colony A and B in Joyce et al.^3^). These populations had high levels of diversity, with detection of all KoRV subtypes except KoRV-E (1 colony out of 2 had KoRV-C)^3^. On average, animals from colony A and B possessed four KoRV subtypes, which is notably higher than the average of two subtypes seen for colonies C-E in this study. Furthermore, whilst detected at low levels in colony A and B^3^, KoRV-C and F were not identified in any of the three colonies investigated in this study. Variation between captive populations is also seen with the most prevalent subtypes, in particular KoRV-B, which was detected in 86.7%, 76.6%, 24.2%, 29.4% and 64.7% of animals housed in colonies A–E, respectively^3^.

Interestingly, the lowest level of KoRV genetic diversity among the three populations analyzed in this study was found in colony C, despite its proximity to the high diversity populations from Joyce, et al.^3^ in SE QLD. This was also the only population to have KoRV-K, a subtype previously detected in colony A and B in SE QLD^3^. This finding suggests that this subtype may currently be localized to this region. Despite their high prevalence of up to 40% in colony A and B however, there was no detection of KoRV subtypes F, G or H in colony C. This large variation in KoRV diversity observed between captive populations is potentially due to historical acquisition of koalas from different sites within Australia. Historical sourcing into these colonies of koalas from geographical areas which have recently been shown to differ greatly in subtype prevalence^11^, may have led to the opportunistic introduction of different KoRV subtypes into these captive populations.

The association of KoRV with koala disease is becoming increasingly more apparent, particularly for the exogenous subtypes^7,14–16,18^. Despite the vast differences in KoRV diversity seen between captive populations within Australia, these institutions do not have any obvious differences in koala life expectancy and health status. This therefore suggests that KoRV may not be as detrimental to captive animals as it might be to wild koalas, which are likely to be exposed to added ecological stressors. However, there are likely potential stressors that are unique to a captive environment. Knowledge of KoRV subtype profiles in koalas across different institutions and their potential links to mortality and morbidity in captive colonies will be important for future breeding and disease management decisions.

This study provides further evidence that exogenous KoRV subtypes frequently transmit from infected dam to joey. This result was significant despite the low diversity and sequence sharing observed in these populations, indicating the strength of this transmission route. These findings support previous results implicating dam-joey transmission as the primary transmission route of exogenous KoRV within SE QLD koalas^3^. Based on the results from this study, we know this transmission route is not unique to those SE QLD colonies and is likely broadly applicable to all koala populations. Whilst not included in our formal analysis, we also found evidence for dam-joey transmission of KoRV-E. This has been documented previously in a US zoo^21^, however, due to the low sample sizes, more comprehensive analyses are required to confirm this finding. Transmission from dam to joey is thought to occur quite readily due to their close proximity and sharing of potentially infectious fluids including milk and pap (semi-fluid faecal matter). Viral transmission through milk and faeces has been documented for other closely related retroviruses^36–38^ and KoRV proteins and RNA have previously been detected in these excretions from koalas^11,39^, indicating KoRV transmission via these routes is possible.

The detection of a KoRV-D sequence within a neonate tissue sample highlights the possibility of in utero KoRV transmission. This neonate failed to make it into its’ mother’s pouch and suckle due to pre-established compromise, with the necroscopy report revealing high levels of amniotic fluid and meconium within the stomach. As expected, no milk was detected. Whilst we cannot rule out the possibility that this KoRV-D sequence is endogenous, another explanation is the mother to joey transmission of this sequence through neonate ingestion of infected amniotic fluid in utero or during parturition. These routes of transmission have been observed previously for GALV^40^. Together, this finding suggests KoRV transmission may also occur during the perinatal period or parturition.

In this study, nine (14.1%) koalas across all three institutions were positive for KoRV-A only. This is comparatively higher than the four KoRV-A positive only animals detected from the 109 (3.7%) SE QLD captive koalas analyzed in our previous study^3^. This finding indicates that the presence of KoRV-A only koalas is more common than previously believed, providing the possibility of reproductive management to produce KoRV-A only animals for captive colonies or for future release back into the wild. Detection of these KoRV-A only animals also provides an opportunity to monitor them for health-related issues in a prospective study to see if subsequent KoRV subtype presentation has any impact on koala health outcomes over time. Given the strong association of exogenous KoRV subtypes with disease onset in koalas^18^, preferentially breeding with KoRV-A only female koalas where possible may be beneficial for colony health as such animals would be expected to produce young that also only carry KoRV-A. This strategy therefore has the potential to reduce the prevalence of exogenous subtypes within the population.

Overall, this study investigated KoRV genetic diversity and transmission dynamics within three Australian captive koala populations. The unique KoRV profiles observed within each population reflect localized transmission dynamics. Supporting previous findings, KoRV transmission was found to occur primarily between dam and joey, likely through the ingestion of milk, pap or other infectious fluids consumed by the joey in utero or during parturition. These findings have significant implications for institutions around the world with captive koala populations, informing appropriate animal management and breeding strategies.

## Supplementary Information

Supplementary Information 1.

Supplementary Information 2.

## Data Availability

Representative sequences for each sequence cluster reported in this paper have been deposited in GenBank and assigned accession numbers ON839090–ON839182.

## References

[1] Hanger JJ (2000). The nucleotide sequence of koala (*Phascolarctos cinereus*) retrovirus: A novel type C endogenous virus related to gibbon ape leukemia virus. J. Virol..

[2] Simmons GS (2012). Prevalence of koala retrovirus in geographically diverse populations in Australia. Aust. Vet. J..

[3] Joyce BA, Blyton MDJ, Johnston SD, Young PR, Chappell KJ (2021). Koala retrovirus genetic diversity and transmission dynamics within captive koala populations. Proc. Natl. Acad. Sci..

[4] Chappell KJ (2017). Phylogenetic diversity of koala retrovirus within a wild koala population. J. Virol..

[5] Sarker N (2019). Genetic diversity of Koala retrovirus env gene subtypes: Insights into northern and southern koala populations. J. Gen. Virol..

[6] Quigley BL (2019). Changes in endogenous and exogenous koala retrovirus (KoRV) subtype expression over time reflects koala health outcomes. J. Virol..

[7] Tarlinton R, Meers J, Hanger J, Young P (2005). Real-time reverse transcriptase PCR for the endogenous koala retrovirus reveals an association between plasma viral load and neoplastic disease in koalas. J. Gen. Virol..

[8] Tarlinton RE, Meers J, Young PR (2006). Retroviral invasion of the koala genome. Nature.

[9] Tarlinton RE (2017). Differential and defective expression of koala retrovirus reveal complexity of host and virus evolution. Biorxiv Preprint.

[10] Legione AR (2017). Koala retrovirus genotyping analyses reveal a low prevalence of KoRV-A in *Victorian koalas* and an association with clinical disease. J. Med. Microbiol..

[11] Blyton MDJ, Young PR, Moore BD, Chappell KJ (2022). Geographic patterns of koala retrovirus genetic diversity, endogenization, and subtype distributions. Proc. Natl. Acad. Sci..

[12] Ávila-Arcos MC (2013). One hundred twenty years of koala retrovirus evolution determined from museum skins. Mol. Biol. Evol..

[13] Ishida Y, Zhao K, Greenwood AD, Roca AL (2015). Proliferation of endogenous retroviruses in the early stages of a host germ line invasion. Mol. Biol. Evol..

[14] McEwen GK (2021). Retroviral integrations contribute to elevated host cancer rates during germline invasion. Nat. Commun..

[15] Fabijan J (2020). Pathological findings in koala retrovirus-positive koalas (*Phascolarctos cinereus*) from northern and southern Australia. J. Comp. Pathol..

[16] Sarker N (2020). Koala retrovirus viral load and disease burden in distinct northern and southern koala populations. Sci. Rep..

[17] Brown AS, Girjes AA, Lavin MF, Timms P, Woolcock JB (1987). Chlamydial disease in koalas. Aust. Vet. J..

[18] Blyton MDJ, Pyne M, Young P, Chappell K (2022). Koala retrovirus load and non-A subtypes are associated with secondary disease among wild northern koalas. PLoS Pathog..

[19] Xu W (2013). An exogenous retrovirus isolated from koalas with malignant neoplasias in a US zoo. Proc. Natl. Acad. Sci..

[20] Shojima T (2013). Identification of a novel subgroup of koala retrovirus from koalas in Japanese zoos. J. Virol..

[21] Xu W, Gorman K, Santiago JC, Kluska K, Eiden MV (2015). Genetic diversity of koala retroviral envelopes. Viruses.

[22] Miyazawa T, Shojima T, Yoshikawa R, Ohata T (2011). Isolation of koala retroviruses from koalas in Japan. J. Vet. Med. Sci..

[23] Johnson RN (2018). Adaptation and conservation insights from the koala genome. Nat. Genet..

[24] Quigley BL, Ong VA, Hanger J, Timms P (2018). Molecular dynamics and mode of transmission of koala retrovirus as it invades and spreads through a wild Queensland koala population. J. Virol..

[25] Hashem MA (2019). Coinfection with koala retrovirus subtypes A and B and its impact on captive koalas in Japanese zoos. Adv. Virol..

[26] Hashem MA (2022). Subtype distribution and expression of the koala retrovirus in the Japanese zoo koala population. Infect. Genet. Evol. J. Mol. Epidemiol. Evol. Genet. Infect. Dis..

[27] Zheng H (2020). Koala retrovirus diversity, transmissibility, and disease associations. Retrovirology.

[28] Quigley BL (2021). Koala retrovirus in northern Australia shows a mixture of stable endogenization and exogenous lineage diversification within fragmented koala populations. J. Virol..

[29] Gillett, A. K. An examination of disease in captive Australian koalas (*Phascolarctos cinereus*) and potential links to koala retrovirus (KoRV). 39–45 (Technical Reports of the Australian Museum, Online, 2014).

[30] Afgan E (2016). The Galaxy platform for accessible, reproducible and collaborative biomedical analyses: 2016 update. Nucleic Acids Res..

[31] Caporaso JG (2010). QIIME allows analysis of high-throughput community sequencing data. Nat. Methods.

[32] Hobbs M (2014). A transcriptome resource for the koala (*Phascolarctos cinereus*): Insights into koala retrovirus transcription and sequence diversity. BMC Genom..

[33] Abts KC, Ivy JA, DeWoody JA (2015). Immunomics of the koala (*Phascolarctos cinereus*). Immunogenetics.

[34] RStudio Team. (RStudio, Inc., 2016).

[35] Hadfield JD (2010). MCMC methods for multi-response generalized linear mixed models: The MCMCglmm R package. J. Stat. Softw..

[36] Kawakami TG, Sun L, McDowell TS (1977). Infectious primate type-C virus shed by healthy gibbons. Nature.

[37] Pacitti AM, Jarrett O, Hay D (1986). Transmission of feline leukaemia virus in the milk of a non-viraemic cat. Vet. Rec..

[38] Gomes-Keller M (2008). Fecal shedding of infectious feline leukemia virus and its nucleic acids: A transmission potential. Vet. Microbiol..

[39] Morris KM (2016). Characterisation of the immune compounds in koala milk using a combined transcriptomic and proteomic approach. Sci. Rep..

[40] Kawakami TG, Sun L, McDowell TS (1978). Natural transmission of gibbon leukemia virus. J. Natl. Cancer Inst..

