# Supplementary material for: Diversity and transmission of koala retrovirus: a case study in three captive koala populations
Source: Sci Rep. 2022 Sep 22;12:15787. doi: 10.1038/s41598-022-18939-6 (PMC9499970; doi:10.1038/s41598-022-18939-6)
Supplement: Supplementary file 1 — Supplementary Information 1. [file 41598_2022_18939_MOESM1_ESM.pdf]

Diversity and transmission of koala retrovirus: a case study in three captive koala populations

Briony A. Joyce[1], Michaela D.J. Blyton[1,2], Stephen D. Johnston[3], William D. Meikle[4†], Kimberly Vinette Herrin[5†], Claire Madden[6†], Paul R. Young[1] and Keith J. Chappell[1,2*]

[1]School of Chemistry and Molecular Biosciences, University of Queensland, St. Lucia, QLD 4072, Australia
[2]Australian Institute for Bioengineering and Nanotechnology, University of Queensland, St. Lucia, QLD 4072, Australia
[3]School of Agriculture and Food Sciences, The University of Queensland, Gatton, QLD 4343, Australia
[4]WILD LIFE Sydney Zoo, Darling Harbour, NSW 2000, Australia
[5]Taronga Wildlife Hospital, Taronga Zoo, Mosman, NSW 2088, Australia
[6]Sea World and Paradise Country, Main Beach, QLD 4217, Australia
[†]These authors have contributed equally to this work

* Correspondence:
Keith Chappell
k.chappell@uq.edu.au

Supplementary File

Table S1

Figure S1

Table S1. Percentage of KoRV reads corresponding to each subtype. Percentage of reads attributable to each subtype is shown for each individual koala housed in both colony C (*n* = 33), colony D (*n* = 17) and colony E (*n* = 17).

Koala	Sample	% of reads grouping with subtype:											
		A	B	C	D	E	F	G	H	I	K	L	M
C1	Blood	99.042	0.000	0.000	0.935	0.000	0.000	0.000	0.000	0.000	0.023	0.000	0.000
C2	Blood	100.000	0.000	0.000	0.000	0.000	0.000	0.000	0.000	0.000	0.000	0.000	0.000
C3	Blood	98.943	0.000	0.000	1.024	0.000	0.000	0.000	0.000	0.000	0.033	0.000	0.000
C4	Blood	97.979	0.000	0.000	1.994	0.000	0.000	0.000	0.000	0.000	0.027	0.000	0.000
C5	Blood	99.046	0.000	0.000	0.935	0.000	0.000	0.000	0.000	0.005	0.014	0.000	0.000
C6	Blood	97.638	0.000	0.000	0.540	0.000	0.000	0.000	0.000	1.821	0.000	0.000	0.000
C7	Blood	99.446	0.040	0.000	0.514	0.000	0.000	0.000	0.000	0.000	0.000	0.000	0.000
C8	Blood	97.648	0.000	0.000	0.930	0.000	0.000	0.000	0.000	1.421	0.000	0.000	0.000
C9	Blood	99.405	0.000	0.000	0.583	0.000	0.000	0.000	0.000	0.000	0.012	0.000	0.000
C10	Blood	99.491	0.000	0.000	0.509	0.000	0.000	0.000	0.000	0.000	0.000	0.000	0.000
C11	Blood	98.930	0.000	0.000	1.070	0.000	0.000	0.000	0.000	0.000	0.000	0.000	0.000
C12	Blood	99.974	0.000	0.000	0.026	0.000	0.000	0.000	0.000	0.000	0.000	0.000	0.000
C13	Blood	99.790	0.000	0.000	0.210	0.000	0.000	0.000	0.000	0.000	0.000	0.000	0.000
C14	Blood	100.000	0.000	0.000	0.000	0.000	0.000	0.000	0.000	0.000	0.000	0.000	0.000
C15	Blood	99.511	0.000	0.000	0.489	0.000	0.000	0.000	0.000	0.000	0.000	0.000	0.000
C16	Blood	98.906	0.000	0.000	1.069	0.000	0.000	0.000	0.000	0.000	0.025	0.000	0.000
C17	Blood	99.008	0.000	0.000	0.880	0.000	0.000	0.000	0.000	0.000	0.112	0.000	0.000
C18	Blood	99.119	0.006	0.000	0.857	0.000	0.000	0.000	0.000	0.000	0.018	0.000	0.000
C19	Blood	96.713	1.556	0.000	1.696	0.000	0.000	0.000	0.000	0.000	0.035	0.000	0.000
C20	Blood	99.224	0.020	0.000	0.736	0.000	0.000	0.000	0.000	0.000	0.020	0.000	0.000
C21	Blood	97.659	0.020	0.000	0.669	0.000	0.000	0.000	0.000	1.652	0.000	0.000	0.000
C22	Blood	99.450	0.014	0.000	0.536	0.000	0.000	0.000	0.000	0.000	0.000	0.000	0.000
C23	Blood	98.314	0.000	0.000	1.301	0.000	0.000	0.000	0.000	0.051	0.334	0.000	0.000
C24	Blood	99.868	0.000	0.000	0.132	0.000	0.000	0.000	0.000	0.000	0.000	0.000	0.000
C25	Blood	98.888	0.000	0.000	1.096	0.000	0.000	0.000	0.000	0.000	0.016	0.000	0.000
C26	Blood	99.742	0.000	0.000	0.258	0.000	0.000	0.000	0.000	0.000	0.000	0.000	0.000
C27	Blood	99.296	0.000	0.000	0.704	0.000	0.000	0.000	0.000	0.000	0.000	0.000	0.000
C28	Blood	97.949	0.000	0.000	1.511	0.000	0.000	0.000	0.000	0.008	0.533	0.000	0.000
C29	Blood	96.674	0.585	0.000	1.019	0.000	0.000	0.000	0.000	1.721	0.000	0.000	0.000
C30	Blood	98.499	0.062	0.000	0.256	0.000	0.000	0.000	0.000	1.184	0.000	0.000	0.000
C31	Blood	99.545	0.000	0.000	0.426	0.000	0.000	0.000	0.000	0.006	0.023	0.000	0.000
C32	Blood	99.751	0.000	0.000	0.220	0.000	0.000	0.000	0.000	0.029	0.000	0.000	0.000
C33	Blood	99.522	0.000	0.000	0.472	0.000	0.000	0.000	0.000	0.000	0.006	0.000	0.000

D1	Blood	99.994	0.000	0.000	0.006	0.000	0.000	0.000	0.000	0.000	0.000	0.000	0.000
D2	Blood	99.108	0.000	0.000	0.892	0.000	0.000	0.000	0.000	0.000	0.000	0.000	0.000
D3	Blood	92.260	7.579	0.000	0.105	0.057	0.000	0.000	0.000	0.000	0.000	0.000	0.000
D4	Blood	95.299	4.413	0.000	0.288	0.000	0.000	0.000	0.000	0.000	0.000	0.000	0.000
D5	Blood	97.943	1.899	0.000	0.074	0.085	0.000	0.000	0.000	0.000	0.000	0.000	0.000
D6	Blood	97.923	1.836	0.000	0.108	0.132	0.000	0.000	0.000	0.000	0.000	0.000	0.000
D7	Blood	95.362	4.602	0.000	0.007	0.029	0.000	0.000	0.000	0.000	0.000	0.000	0.000
D8	Blood	100.000	0.000	0.000	0.000	0.000	0.000	0.000	0.000	0.000	0.000	0.000	0.000
D9	Blood	99.479	0.000	0.000	0.521	0.000	0.000	0.000	0.000	0.000	0.000	0.000	0.000
D10	Blood	100.000	0.000	0.000	0.000	0.000	0.000	0.000	0.000	0.000	0.000	0.000	0.000
D11	Blood	100.000	0.000	0.000	0.000	0.000	0.000	0.000	0.000	0.000	0.000	0.000	0.000
D12	Blood	99.269	0.000	0.000	0.731	0.000	0.000	0.000	0.000	0.000	0.000	0.000	0.000
D13	Blood	100.000	0.000	0.000	0.000	0.000	0.000	0.000	0.000	0.000	0.000	0.000	0.000
D14	Blood	100.000	0.000	0.000	0.000	0.000	0.000	0.000	0.000	0.000	0.000	0.000	0.000
D15	Neonate	99.983	0.000	0.000	0.017	0.000	0.000	0.000	0.000	0.000	0.000	0.000	0.000
D16	Neonate	100.000	0.000	0.000	0.000	0.000	0.000	0.000	0.000	0.000	0.000	0.000	0.000
D17	Spleen	98.471	0.000	0.000	1.529	0.000	0.000	0.000	0.000	0.000	0.000	0.000	0.000
E1	Blood	98.376	0.000	0.000	0.182	0.000	0.000	0.000	0.000	1.442	0.000	0.000	0.000
E2	Blood	96.438	1.144	0.000	0.380	0.000	0.000	0.000	0.000	2.037	0.000	0.000	0.000
E3	Blood	97.828	0.880	0.000	0.000	0.000	0.000	0.000	0.000	1.292	0.000	0.000	0.000
E4	Blood	96.967	0.000	0.000	0.010	0.000	0.000	3.024	0.000	0.000	0.000	0.000	0.000
E5	Blood	82.265	0.091	0.000	0.115	0.000	0.000	0.000	0.000	17.529	0.000	0.000	0.000
E6	Blood	99.440	0.000	0.000	0.352	0.000	0.000	0.000	0.207	0.000	0.000	0.000	0.000
E7	Blood	96.518	1.857	0.000	0.098	0.000	0.000	0.000	0.000	1.527	0.000	0.000	0.000
E8	Blood	97.058	2.024	0.000	0.000	0.000	0.000	0.000	0.000	0.918	0.000	0.000	0.000
E9	Blood	98.887	0.654	0.000	0.389	0.000	0.000	0.000	0.070	0.000	0.000	0.000	0.000
E10	Blood	100.000	0.000	0.000	0.000	0.000	0.000	0.000	0.000	0.000	0.000	0.000	0.000
E11	Blood	97.115	2.142	0.000	0.015	0.000	0.000	0.000	0.000	0.728	0.000	0.000	0.000
E12	Blood	98.234	0.450	0.000	0.008	0.000	0.000	0.000	0.000	1.309	0.000	0.000	0.000
E13	Blood	94.754	2.247	0.000	0.602	0.000	0.000	0.000	0.000	2.398	0.000	0.000	0.000
E14	Blood	100.000	0.000	0.000	0.000	0.000	0.000	0.000	0.000	0.000	0.000	0.000	0.000
E15	Blood	99.572	0.000	0.000	0.428	0.000	0.000	0.000	0.000	0.000	0.000	0.000	0.000
E16	Blood	98.720	1.122	0.000	0.156	0.000	0.000	0.000	0.000	0.002	0.000	0.000	0.000
E17	Blood	97.516	1.528	0.000	0.573	0.000	0.000	0.000	0.000	0.383	0.000	0.000	0.000

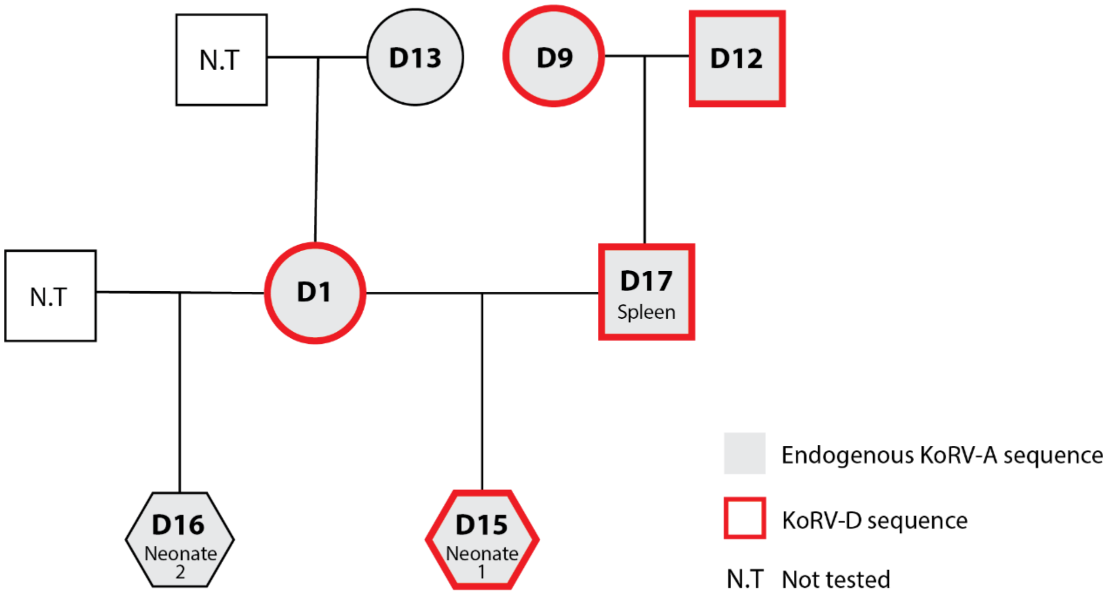

Figure S1. KoRV sequences identified in one koala family cluster from colony D. Sequences detected in genomic DNA from PBMCs within whole blood or within tissue samples (spleen or neonate, as indicated). Females and males are denoted by circles and squares, respectively. Hexagons represent neonate samples without a defined sex.
